# Facial Ligament Thickening Using Poly‐D,L‐Lactic Acid Injection

**DOI:** 10.1111/jocd.70300

**Published:** 2025-06-26

**Authors:** Kyu‐Ho Yi, Benrita Jitaree, Song Eung Yoon, Isabella Rosaline, Deborah Chua, Jovian Wan, Ruri Pamela, Hyun‐Jin Park, Soo‐Bin Kim

**Affiliations:** ^1^ Division in Anatomy and Developmental Biology, Department of Oral Biology, Human Identification Research Institute, BK21 FOUR Project Yonsei University College of Dentistry Seoul Korea; ^2^ You and I Clinic Seoul Korea; ^3^ Chakri Naruebodindra Medical Institute, Faculty of Medicine Ramathibodi Hospital Mahidol University Bangkok Thailand; ^4^ BRANDNEW Aesthetic Surgery Clinic Seoul Korea; ^5^ Avery Beauty Clinic and Avena Aesthetics Indonesia Jakarta Indonesia; ^6^ Solitaire Aesthetics Singapore Singapore; ^7^ Medical Research Inc. Wonju Korea; ^8^ CELV Dermatology Clinic Jakarta Indonesia; ^9^ Department of Anatomy, School of Medicine Konkuk University Chungju Republic of Korea; ^10^ Department of Oral Anatomy, institute of Biomaterial Implant, College of Dentistry Wonkwang University Iksan South Korea

**Keywords:** facial ligaments, facial rejuvenation, poly‐D,L‐lactic acid, ultrasonography, zygomatic ligament

## 1 Introduction

Facial aging is a multifactorial process influenced by various structural and physiological changes. These include the loss of skeletal support, elongation of facial ligaments, redistribution and descent of fat compartments, thinning of the superficial muscular aponeurotic system (SMAS) layer, and atrophy of subcutaneous and dermal tissues [1]. Together, these changes lead to the descent of facial structures and the development of wrinkles. Of particular importance is the elongation of true facial ligaments, which plays a critical role in the downward displacement of soft tissues, contributing significantly to the sagging and aged appearance of the face [2].

Currently, various polymers have been investigated for their ability to provide subtle, long‐lasting rejuvenation effects. Among these, poly‐D,L‐lactic acid (PDLLA) has emerged as a promising option. This novel soft tissue filler is characterized by its biocompatibility, biodegradability, and biostimulatory properties. Its primary mechanism involves addressing soft tissue volume loss through the stimulation of collagen synthesis within the dermis [3, 4, 5, 6, 7]. While numerous studies have demonstrated the efficacy of PDLLA in skin rejuvenation, the treatment of stretch marks, and the repair of acne scars, research focusing on its effects on facial ligaments remains limited. This study seeks to assess the efficacy and safety of PDLLA injections targeted at the zygomatic ligament and to explore their potential impact on facial rejuvenation.

## 2 Clinical Case

**FIGURE 1:**
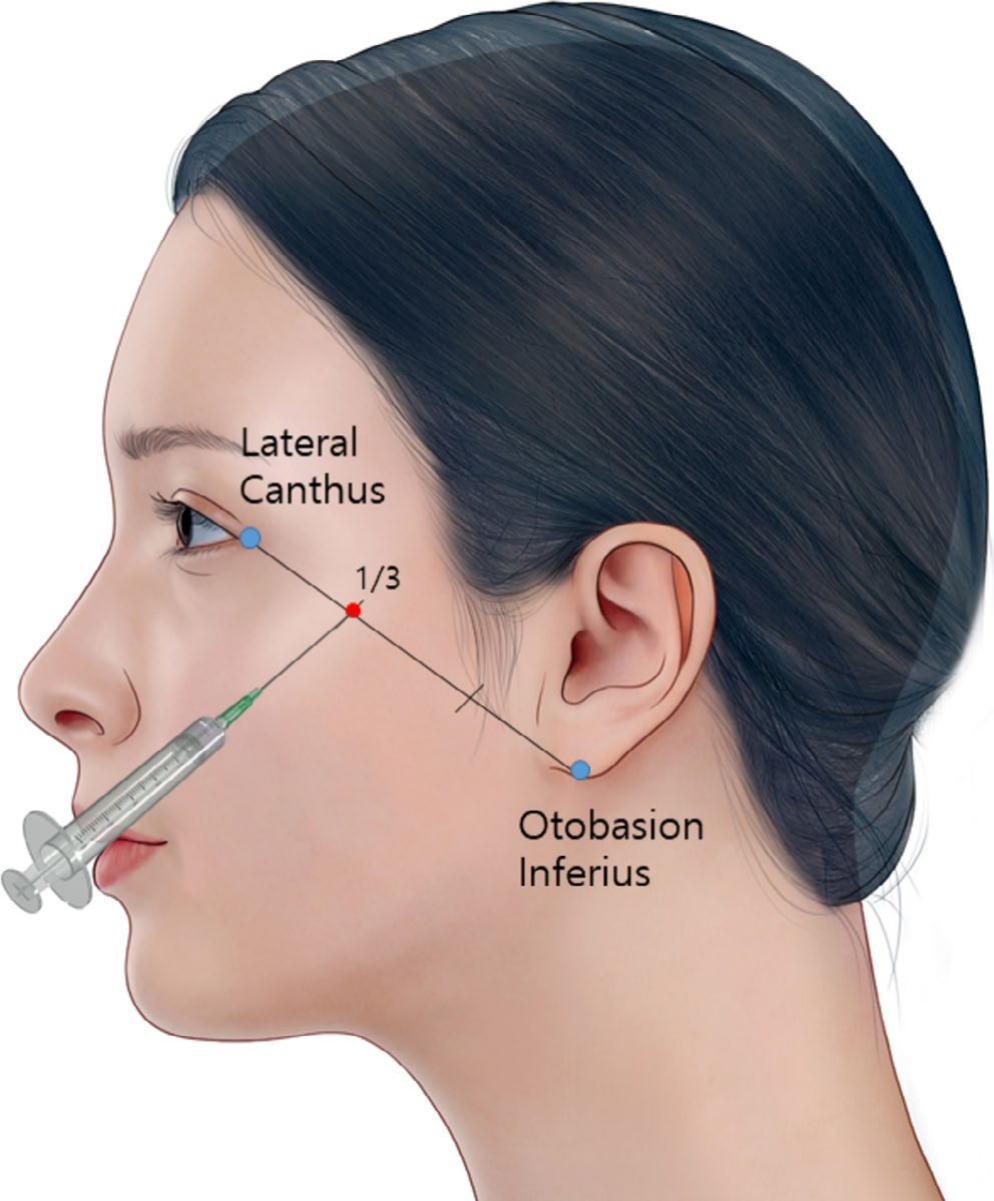
Injection technique targeting the zygomatic ligament. The injection point is marked at the one‐third distance along the line connecting the lateral canthus to the otobasion inferius. A 3 mL retrograde injection was administered at a 90‐degree angle during each of four treatment sessions. No adverse reactions were observed throughout the treatment period.

A female patient with mild gravitational ptosis affecting both cheeks due to aging underwent bilateral injections of PDLLA. The PDLLA product used in this treatment was Juvelook Volume (Lenisna, VAIM Inc., Seoul, Korea), with each vial containing 170 mg of PDLLA and 30 mg of noncross‐linked hyaluronic acid (HA). The particle size of the filler ranged between 40 and 60 μm. To prepare the product, one vial was mixed with 9 mL of normal saline 1 day prior to the procedure. On the treatment day, the vial was shaken using a vortex mixer for 30 min before administration. The patient underwent four treatment sessions, with 3 mL of the product injected into the zygomatic ligament during each session. No adverse reactions were reported throughout the treatment period. The injection point was located at the one‐third mark along the line from the lateral canthus to the otobasion inferius, with a retrograde injection of 3 mL performed (Figure 1). Each 3 mL injection was performed in a linear retrograde manner using a 23G needle inserted perpendicular (90°) to the skin and withdrawn slowly to evenly distribute the filler along the zygomatic ligament. Postinjection molding was conducted to prevent localized swelling or lump formation.

**FIGURE 2:**
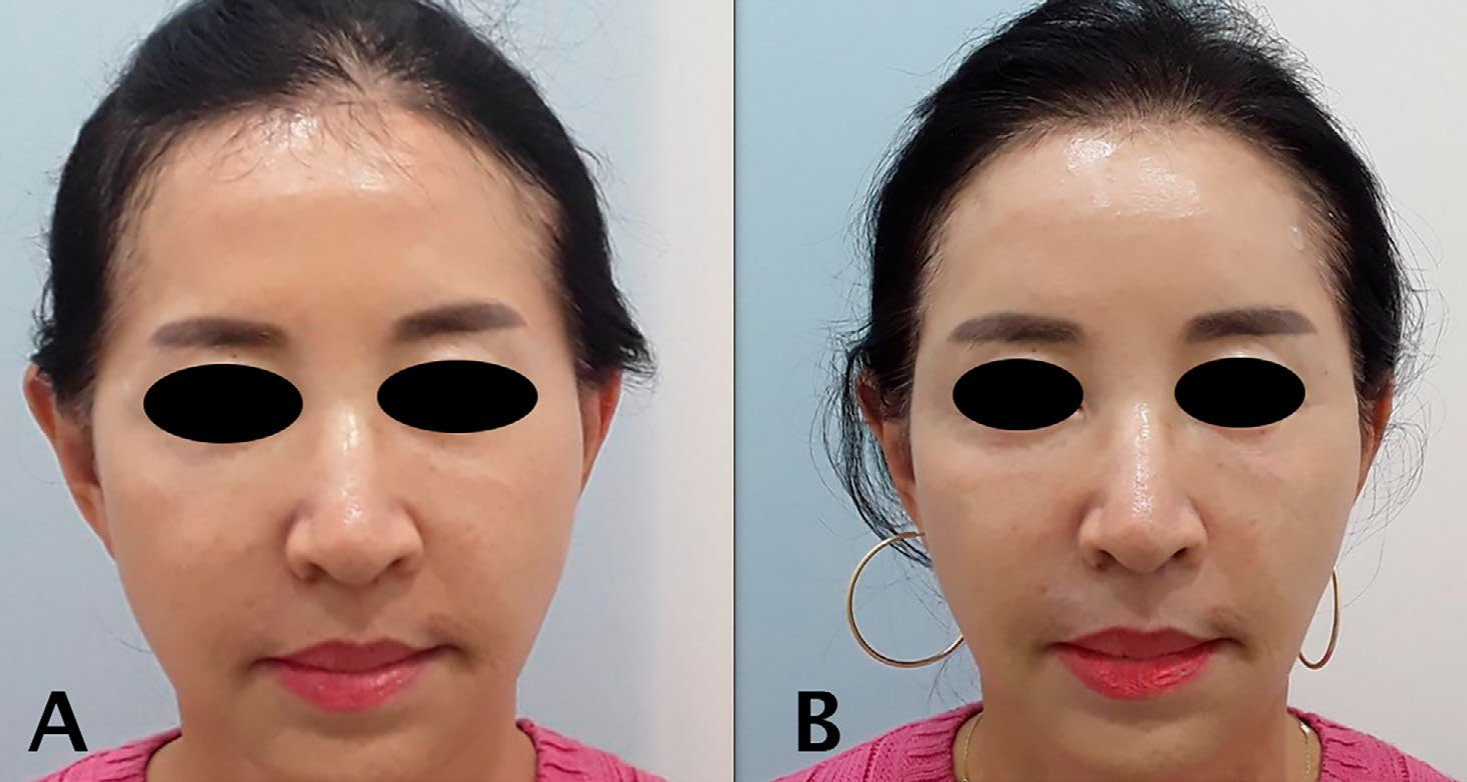
The patient before (A) and after (B) four sessions of PDLLA treatment.

**FIGURE 3:**
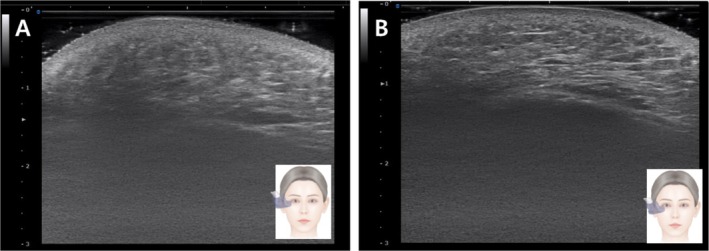
Horizontal ultrasonographic image of the zygomaticus ligament proper before (A) and after (B) treatment.

**FIGURE 4:**
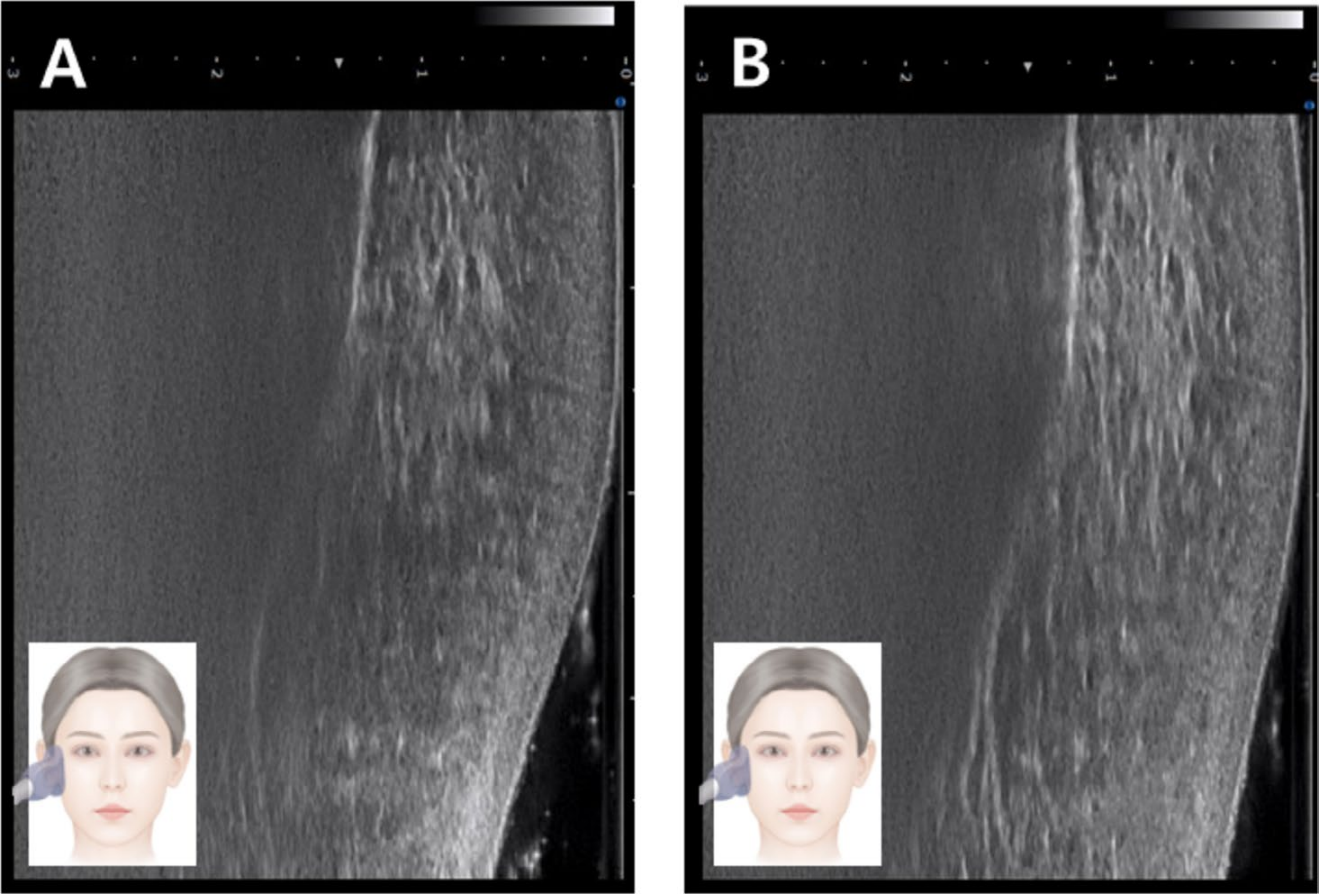
Vertical ultrasonographic image of the zygomaticus ligament proper before (A) and after (B) treatment.

Standardized clinical photographs and ultrasound images were taken at baseline and at follow‐up appointments to monitor outcomes. All imaging was conducted under controlled lighting and patient positioning within a designated photography space. Posttreatment images were assessed separately by the physician and the patient using the Global Aesthetic Improvement Scale (GAIS). The patient reported high satisfaction, with a GAIS score of 3 (Figure 2). Ultrasound examination conducted 3 weeks after the final treatment revealed structural changes, with the zygomatic ligament appearing denser and thicker (Figures 3 and 4).

## 3 Discussion

One of the most prominent and impactful aspects of facial aging is the elongation of facial ligaments, which contributes significantly to the downward displacement of facial structures [1]. Detailed anatomical analysis underscores the critical role of true retaining ligaments in maintaining the structural integrity of the face. These ligaments consist of fibrous bands that extend from the periosteum through the SMAS to the subcutaneous tissue, supporting the overlying facial layers [2].

There are four primary true retaining ligaments responsible for the structural support of the face: the orbital retaining ligament (ORL), zygomatic retaining ligament, buccal‐maxillary retaining ligament, and mandibular retaining ligament. Among these, the zygomatic ligament plays a particularly vital role in facial aging due to its location [8]. This ligament supports a substantial area of the mobile subcutaneous tissues of the face, making it a frequent focus for studies aiming to develop alternative approaches to facial rejuvenation [9].

The zygomatic cutaneous ligaments are composed of robust fibers originating from the inferior border of the zygomatic arch and extending anteriorly to the junction of the zygomatic arch and body [9, 10]. These fibers often appear as fibrous septa along the posterior aspect of the arch and can take on a cylindrical form near the origin of the zygomaticus major muscle [9, 10, 11]. The zygomatic ligaments are classified as true ligaments due to their direct attachment to the dermis [9, 10]. Mendelson et al. identified the position of the zygomatic ligaments medial to the junction of the arch and body, aligned with the origins of key facial expression muscles, including the zygomaticus major, zygomaticus minor, and levator labii superioris [12, 13].

The elongation of true ligaments results in the downward displacement of overlying tissues, leading to visible sagging of the skin. Consequently, anti‐aging treatments should focus on retightening these retaining ligaments to achieve a lifting effect. Strengthening the ligaments indirectly elevates the SMAS layer, contributing to facial rejuvenation.

Several methods can effectively support facial ligaments, including injectable treatments such as hyaluronic acid and poly‐lactic acid and its isomers. PDLLA, a polymer formed through the copolymerisation of L‐lactic acid and D‐lactic acid or their lactide derivatives, has shown particular promise. When these components are combined in a 1:1 ratio, PDLLA forms an amorphous material with a glass transition temperature of 50°C to 60°C [3].

The safety and efficacy of PDLLA have been evaluated in numerous clinical trials. PDLLA has demonstrated excellent tolerability and safety across various surgical and dermatological applications. Studies have shown its effectiveness in improving wound healing, reducing fine lines and wrinkles, and enhancing the appearance of scars [4, 5]. While prior research has primarily focused on its applications for skin rejuvenation and regeneration, its effects on ligament integrity remain underexplored.

Poly‐D,L‐lactic acid is a biocompatible and biodegradable polymer. The injectable PDLLA used in this study consisted of 170 mg of PDLLA and 30 mg of noncrosslinked hyaluronic acid (HA) per vial. The microparticles, which range in size from 30 to 70 μm, are small enough to pass through injection needles or cannulas but large enough to resist phagocytosis by skin cells [6, 7, 14, 15, 16]. These microparticles exhibit a spongiform microsphere structure [7].

There is growing evidence supporting the efficacy of PDLLA in ligament regeneration. An in vitro study demonstrated significant chondrocyte and mesenchymal stem cell growth on PLA scaffolds, indicating the potential of PLA nanofibres for ligament repair [17]. Additionally, Sarukawa and colleagues observed enhanced adhesion, proliferation, and extracellular matrix production in anterior cruciate ligament fibroblasts when PLA fibers were coated with chitosan [18]. Other studies have further confirmed the role of PLA in promoting fibroblast attachment and proliferation [19].

Injectable PDLLA is supplied as a lyophilised powder, reconstituted with 9 mL of normal saline, as recommended by the manufacturer. Reconstitution was performed using a vortex mixer for approximately 30 min. In this study, the product was injected using a linear retrograde technique with a 23G needle, targeting the zygomatic ligament at the supraperiosteal plane. The patient underwent four treatment sessions at three‐week intervals, with 3 mL of PDLLA administered per session. Ultrasonographic examinations of the zygomatic region were performed pre and posttreatment, focusing on the zygomatic ligament. The findings revealed a more hyperechoic and thickened ligament structure postinjection (Figure 2A,B), while the ligament's smooth contour and linearity remained consistent.

This study demonstrated noticeable improvements in ligament thickness and clinical outcomes following PDLLA injections. The unique rheological and biostimulatory properties of PDLLA, combined with precise placement beneath a lax zygomatic ligament, effectively addressed ligament laxity and stimulated neocollagenesis simultaneously. The procedure proved to be an effective intervention for correcting age‐related changes in patients with mild to moderate gravitational ptosis. The patient reported high satisfaction, describing a lifting and supportive effect on her cheeks without excessive facial volume. No significant adverse events related to the procedure were observed.

However, the study has several limitations. The short follow‐up period hindered the assessment of long‐term outcomes. Future research with larger sample sizes and extended follow‐up durations would provide more robust evidence. Additional studies incorporating objective evaluation methods, such as 3D imaging and histological analysis, are also needed to confirm these findings. Investigations into the optimal dilution ratio for PDLLA are warranted to maximize efficacy.

In conclusion, this study provides compelling evidence that PDLLA injections can strengthen facial ligaments and deliver an indirect lifting effect on overlying tissues, offering a promising approach to facial rejuvenation.

## Author Contributions

All authors have reviewed and approved the article for submission. Jovian Wan, Soo‐Bin Kim, Song Eun Yoon; Kyu‐Ho Yi: conceptualization. Jovian Wan, Soo‐Bin Kim, Song Eun Yoon, Ruri Pamela, Benrita Jitaree, Isabella Rosaline, Deborah Chua: writing – original draft preparation. Jovian Wan, Soo‐Bin Kim, Song Eun Yoon: writing – review and editing. Jovian Wan, Kyu‐Ho Yi: visualization. Kyu‐Ho Yi: supervision.

## Consent

Informed consent was obtained from all participants, with full disclosure of the study's purpose, risks, and confidentiality.

## Conflicts of Interest

The authors declare no conflicts of interest.

## Data Availability

Research data are not shared.
